# Using a quality improvement approach to improve maternal and neonatal care in North Kivu, Democratic Republic of Congo

**DOI:** 10.1080/09688080.2017.1403276

**Published:** 2017-12-12

**Authors:** Michelle Hynes, Kate Meehan, Janet Meyers, Leon Mashukano Maneno, Erin Hulland

**Affiliations:** a Epidemiologist, U.S. Centers for Disease Control and Prevention, Atlanta, GA, USA; b Health Scientist, U.S. Centers for Disease Control and Prevention, Atlanta, GA, USA; c Deputy Director Health Policy and Practice (Former), International Medical Corps, Washington, DC, USA; d Reproductive Health Manager, International Medical Corps, Goma, Democratic Republic of Congo; e Statistician, U.S. Centers for Disease Control and Prevention, Atlanta, GA, USA

**Keywords:** maternal and neonatal health care, humanitarian conflict, the Democratic Republic of Congo, quality improvement, participatory approaches, global health security

## Abstract

Providing quality health care services in humanitarian settings is challenging due to population displacement, lack of qualified staff and supervisory oversight, and disruption of supply chains. This study explored whether a participatory quality improvement (QI) intervention could be used in a protracted conflict setting to improve facility-based maternal and newborn care. A longitudinal quasi-experimental design was used to examine delivery of maternal and newborn care components at 12 health facilities in eastern Democratic Republic of Congo. Study facilities were split into two groups, with both groups receiving an initial “standard” intervention of clinical training. The “enhanced” intervention group then applied a QI methodology, which involved QI teams in each facility, supported by coaches, testing small changes to improve care. This paper presents findings on two of the study outcomes: delivery of active management of the third stage of labour (AMTSL) and essential newborn care (ENC). We measured AMTSL and ENC through exit interviews with post-partum women and matched partographs at baseline and endline over a 9-month period. Using generalised equation estimation models, the enhanced intervention group showed a greater rate of change than the control group for AMTSL (aOR 3.47, 95% CI: 1.17–10.23) and ENC (OR: 49.62, 95% CI: 2.79–888.28), and achieved 100% ENC completion at endline. This is one of the first studies where this QI methodology has been used in a protracted conflict setting. A method where health staff take ownership of improving care is of even greater value in a humanitarian context where external resources and support are scarce. DOI: 10.1080/09688080.2017.1403276

## Introduction

Health care delivery in humanitarian settings, and subsequent morbidity and mortality, are affected directly and indirectly through armed conflict. Providing health care in humanitarian settings contributes to global health security by managing public health threats at their source. Population movements, breakdowns in health care infrastructure, disruption of supply chains, and lack of health care staff and supervision are all challenges in providing quality health care.^1^ Internally displaced persons (IDPs) and host communities may suffer even greater excess mortality than refugee populations.^2^ In humanitarian crises, little attention has been given to evaluating the efficacy and quality of sexual and reproductive health (SRH) interventions, particularly in protracted conflict settings. According to a review of the evidence for interventions in humanitarian crises, more research is needed focusing on the quality of health service delivery packages.^3^ The Minimum Initial Service Package (MISP) was established by the Inter-Agency Working Group for Reproductive Health in Crises (IAWG) as a minimum set of priority activities and high-impact interventions to be undertaken in a coordinated manner by trained staff during the onset of an emergency, with expanded activities over time.^4^

Active management of the third stage of labour (AMTSL) is identified by the World Health Organization (WHO) as a critical intervention to prevent post-partum haemorrhage, one of the leading causes of maternal mortality.^5^ Essential newborn care (ENC) is a set of interventions that should be provided to all newborns even in an acute phase of a humanitarian emergency and includes thermal care, infection prevention, feeding support, monitoring for danger signs, and postnatal care checks.^6^ IAWG members have prioritised basic emergency obstetric and neonatal care (BEmONC), including AMTSL, and ENC as areas which require further research on effective programming and implementation in emergency settings.^7^ This paper contributes to these critical issues by examining the provision of maternal and newborn care and whether care delivery is improved through a participatory quality improvement (QI) process at health facilities serving conflict-affected populations in North Kivu, Democratic Republic of the Congo (DRC).

### Protracted violence in the DRC

The DRC has experienced protracted conflict for the past two decades, within its borders and as a result of neighbouring conflicts. Political insecurity has further increased the violence and in 2016, DRC had the highest number of new conflict-related IDPs globally.^8^ The United Nations estimates that as of December 2016, there were 3.7 million IDPs in DRC.^9,10^ Most IDPs in DRC have been displaced due to violence in the east, which includes North Kivu Province. North Kivu hosts 23.3% of IDPs in DRC, and approximately 82% of the 863,000 IDPs in North Kivu province live with host families.^10^ Host families often share food, housing, and farm land with their guests, creating an additional burden and strain on their resources.

### Maternal and neonatal health in DRC

Nationally in DRC, a woman has a 1 in 24 lifetime risk of dying from maternal causes and over 30 newborn deaths will occur out of 100,000 live births.^11^ Outcomes are poor in North Kivu; the maternal mortality ratio for the first half of 2013 was 790 deaths per 100,000 live births.^12^ Insecurity, poor health care infrastructure, lack of BEmONC, and delays in seeking, reaching, and receiving care are just some of the causes for such high mortality. The percentage of deliveries attended by skilled birth attendants in the study health zones ranges from 42% to 62% of expected deliveries and the percentage of women completing four antenatal care visits is under 20% in some areas.^13^ Accessing health facilities with BEmONC capacity can take over two days by foot for some communities in North Kivu.

## Methods

### Study design

This paper presents findings on maternal and newborn care delivery as part of a larger pilot study that used a longitudinal, quasi-experimental mixed methods study design to evaluate the implementation of components of MISP and BEmONC using a QI approach. The project was a collaboration between International Medical Corps (IMC), University Research Co., LLC (URC), US Centers for Disease Control and Prevention (CDC), United Nations Population Fund (UNFPA), and the DRC Ministry of Health, North Kivu Province (MoH). IMC has been working in DRC since 1999, supporting over two million people, 80% of whom are displaced. IMC collaborates closely with the MoH to support clinics and hospitals in North and South Kivu by providing medical supplies, training for health workers and referral services. The study areas were selected based on where IMC was providing such support in North Kivu.

A convenience sample of 12 health facilities, where IMC had ongoing programmatic activities, were selected from 3 of 34 health zones of North Kivu province including 6 in Itebero health zone, 3 in Walikale health zone, and 3 in Kibua health zone. Of the 12 health facilities, 10 are primary care health facilities that provide services for uncomplicated deliveries and 2 are referral health facilities with emergency obstetric care capacity. All facilities serve IDP and host populations.

A baseline evaluation conducted over a six-week period on aspects of maternal and neonatal care delivery was conducted at all study facilities prior to any intervention activities, through interviews with post-partum women and data from matched partographs. Healthcare staff working in labour and delivery from the 12 study facilities received an initial “standard” intervention of clinical care training in BEmONC and ENC by IMC and the MoH, which included training on filling out partographs. The training was a total of 12 days and consisted of 4 days of theoretical sessions, 4 days of practical applications on anatomical models, 3 days of practice in health facilities, and 1 day of general synthesis. In addition, all study facilities were provided with new equipment and medical supplies contained in UNFPA’s humanitarian reproductive health kits.

Study facilities were split into two groups based on geographical location. The “enhanced” intervention group was assigned to participate in the QI intervention, which involved training teams in each facility to test small changes to improve care. Facilities were matched as much as possible between groups on the following characteristics in order to ensure comparability of findings: type of facility and level of care available (each group had five primary health facilities with BEmONC capacity and one referral level health facility capable of providing EmONC), size of the population served, and average number of deliveries per month.

The QI intervention was implemented for nine months, after which an endline evaluation was conducted over a six-week period. The control group received training on the QI methodology after endline data collection was completed.

### Ethics approval

Ethics review and approval was obtained by the Ethics Committee of the Université Libre des Pays des Grands Lacs in Goma, DRC; the CDC Human Subjects Research Office judged CDC staff were not engaged in human subjects research as the technical assistance provided did not involve human subject interaction or analyses of identifiable data. Verbal informed consent was obtained from all participants prior to data collection.

### Enhanced intervention

The *Model for Improvement*, developed by University Research Company (URC), is a participatory QI approach that has been successfully applied in a wide variety of health care settings in low- and middle-income countries to improve maternal and child health, care for HIV/AIDS and tuberculosis patients, and for vulnerable children, among others, at the facility and community level.^14–16^ An improvement approach has been used successfully in a humanitarian setting to improve facility-and community-based maternal and newborn care in Afghanistan, in which 3 of the 10 provinces in the study were experiencing protracted conflict.^17^ This project saw increases in the correct use of partographs, the proportion of women who knew maternal and newborn danger signs, and the proportion of facilities who correctly delivered AMSTL over a number of years during the project. A strength of this QI methodology is the ownership of the process by those who know the health system best. Health facility staff can best identify the strengths and weaknesses where they work and devise actions to improve that system. Our study provides an opportunity to explore the application of the QI approach in an exclusively protracted conflict setting in another part of the world.

For the study QI intervention, URC trained healthcare teams from each enhanced intervention facility in QI. These teams consisted of health facility staff who were involved in maternal and neonatal care at multiple levels (for example, facility gatekeeper, registrar, and midwife). As part of the participatory process, each team selected actions they felt could improve some aspect of maternal and newborn care delivery. The teams tested one action at a time, collected process data during the testing period, and then used the process data to assess whether the action resulted in care improvement. If deemed successful, actions were adopted. Unsuccessful actions were discussed by the team who then either modified or developed a new action to address the improvement objectives. Examples of actions tested by the QI teams include ensuring the availability of equipment and supplies in the delivery room so that care was delivered in a timely manner even with one birth attendant. Other actions dealt with reinforcing clinical skills covered in the training, or training staff who had not attended the clinical training. The teams continued with implementing and testing actions throughout the intervention period. IMC and MoH staff, who provide supervision to health facilities as part of their regular work, received additional training from URC in how to support and coach improvement teams. Coaches visited the facilities every one to two months to provide guidance and review process data and progress achieved with the teams. URC and IMC provided continuous feedback and guidance.

### Study data sources

Analyses were conducted with the following two sources of data collected during baseline (November–December, 2015) and endline (September–November, 2016). Face-to-face patient exit interviews (PEI) were conducted with women in the study health facilities who had had a spontaneous vaginal delivery without complications for either the mother or newborn during the two data collection periods. Interviewers were women from the communities served by the study health facilities and trained by the IMC research team over eight days. Training included pilot testing of the questionnaire and adjustments to questionnaire items based on the testing. The questionnaire included demographics, pregnancy history, care delivered to mother and newborn during labour and delivery, and perceived quality of care. The questionnaire was translated from French to Swahili and back-translated before being piloted. Women were eligible to participate in the exit interviews if they were at least 18 years of age, had a normal spontaneous vaginal delivery (without complications for mother or baby) at one of the study facilities, spoke Swahili, and had the mental capacity to give informed consent. Complications that excluded women from the study included: caesarian section, haemorrhage, transfer to a higher level of care for mother or newborn, and newborn asphyxia. Women who needed an episiotomy were not excluded. Completed MoH partographs (graphical records of maternal and foetal information during labour, and maternal and newborn care during delivery and post-partum) were matched to PEIs. The IMC research team collected the matched partograph information following the interviews.

### Sample size calculations

The sample size was calculated based on the number of women needed for the exit interviews to measure our study indicators via two separate cross-sectional samples at baseline and endline. Because prevalence rates are unknown, the research team assumed the most conservative estimate of 50% prevalence of all study indicators. Assuming power of 80% and an alpha of 0.05, a sample size of 97 per group was needed to detect an absolute difference of 20% for the maternal and neonatal outcomes between baseline and endline. Anticipating a non-response rate of 10%, a sample of 107 women per group per time point for a total of 214 women at each time point and 428 women overall was required.

### Measures

As maternal and neonatal mortality are rare events, there was not sufficient statistical power to identify significant changes in these outcomes. Therefore, the proxy indicators of AMSTL and ENC were used to measure changes *within* and *between* groups over time.

For the purpose of this study, AMSTL was defined as having two elements completed: delivery of an uterotonic drug (in DRC, the recommended drug is oxytocin) and uterine massage after delivery of the placenta (Table 1). These components were assessed by self-report from the PEIs. Controlled cord traction, often included as a component of AMTSL, was not included in this study as the WHO only recommends controlled cord traction where skilled birth attendants are present and have received specialised care, which was not always the case for the study facilities.

There are multiple actions that are essential to quality newborn care. For this study, three key actions were assessed to determine whether essential newborn care was provided: (1) weighing of the newborn, (2) application of tetracycline to the newborn’s eyes, and (3) clean cord care (Table 1). These actions were selected based on their inclusion on partographs. Data from PEIs was not used to measure newborn care, as it was possible the care occurred out of sight of the mother.

The following sociodemographic measures were included in the analyses as categorical variables: age (18–24, 25–34, 35+), marital status (single/non-cohabitating, married/cohabitating), parity (primiparous, multiparous), and schooling (none, primary, secondary, or higher).

### Analyses

All analyses were performed using SAS version 9.3. Descriptive statistics summarised demographic and clinical characteristics between baseline and endline and between both groups. Chi-square and *t*-tests were used to assess for statistical significance. *T*-tests were conducted within each group for the AMTSL and ENC outcomes to determine whether there was a significant change over time. Furthermore, differences between groups at baseline were also assessed via *t*-tests to determine if the groups were starting out with varying levels of care.

In order to assess the impact of the QI intervention on AMTSL outcomes, generalised estimating equations (GEE) were used considering time (baseline to endline), group (whether or not the site received the QI), and an interaction between time and group to assess the difference-in-differences – rate of change – across the two groups. Each model accounted for repeated within-clinic measurements using a conservative exchangeable correlation matrix. Two different models were created: one looking at just the influence of the QI intervention over time, and a second controlling for demographic factors, including age group, educational status, marital status, and parity. Multicollinearity between demographics was assessed via correlations and variance inflation factors (VIFs).

The GEE model to assess ENC rate of change was modified due to the enhanced intervention group reaching 100% care delivery at endline. To account for the zero cell count, and therefore no variability, the Haldane correction of 0.5 was added to each cell using aggregated data for time, group, and rate of change only.^18^

## Results

Baseline data collection was conducted between 6 November and 22 December 2015 and endline data collection occurred from 22 September to 10 November 2016. Endline data collection was moved up by one month in order to avoid anticipated violence surrounding national elections scheduled for late November 2016, and thus shortened the intervention period slightly.

### Health facility characteristics

Information on the facilities and staff was collected at baseline. Facilities reported having between two and six providers that were able to oversee deliveries (mean = 3.5). Providers were trained birth attendants, nurses, and nurse midwives. Twenty-three health providers that worked in the maternity wards were interviewed (2 from each of 11 facilities and 1 from the remaining facility) on their maternal and newborn training and knowledge of danger signs for pregnant women and newborns.

Twelve of the 23 providers (52.1%) had received training on normal deliveries (without complications), while 10 (43.4%) had received training on delivery complications. All of the trainings had taken place since 2010 and most were provided by IMC. Only six providers (26.0%) responded that they had received training on AMSTL.

Eight providers (34.7%) received training in essential newborn care in the previous five years and the majority were provided by IMC. Seven of the eight providers who had received essential newborn care training also reported having received training on newborn complications and one additional provider had not been trained on essential newborn care but had received training on newborn complications. Eighteen providers (78.2%) responded that they knew the 9 danger signs for pregnant women but only 11 (47.8%) providers were able to name 4 of them. Sixteen providers (69.5%) responded that they knew the danger signs for newborns, but only nine (39.1%) were able to correctly name four danger signs.

During the baseline data collection period, 257 interviews were completed, 142 in the enhanced intervention group, and 115 in the control group, among women who had vaginal births without complications. There were no refusals, and 200 (78%) interviews were matched to a completed partograph. During the endline data collection period, 224 interviews were completed, 133 in the enhanced intervention group, and 91 in the control group, and 194 (87%) partographs were matched to interviewed women.

The PEI contained questions about displacement status and use of health facilities for deliveries (Table 2). Among interviewed women who had matched partographs, 12.0% at baseline and 43.3% at endline were self-identified as displaced. Of those, 62.5% and 46.4% had been displaced for two years or less at baseline and endline, respectively. Approximately 80% of women arrived at the health facility by foot and 86% stated that they wanted to deliver at that health centre, for both time periods. Among women who had a prior pregnancy, 54.1% at baseline and 48.0% at endline had delivered at the same health facility for the most recent delivery. About 7% of women had delivered a prior pregnancy at home for both time periods (Table 2).

### Sociodemographic characteristics

Sociodemographic characteristics of PEI participants by time point are shown in Table 3. In total, 394 women, 18 years or older, who had recently delivered at one of the study health facilities and had matched partographs, participated in this study; 200 at baseline and 194 at endline. There were no significant differences in sociodemographic characteristics between baseline and endline participants, except for mean age, which was slightly higher at 26.8 years compared with a baseline mean age of 25.3 years (*p* = .02).

### Rate of change in AMTSL and ENC between groups

**Figure 1:**
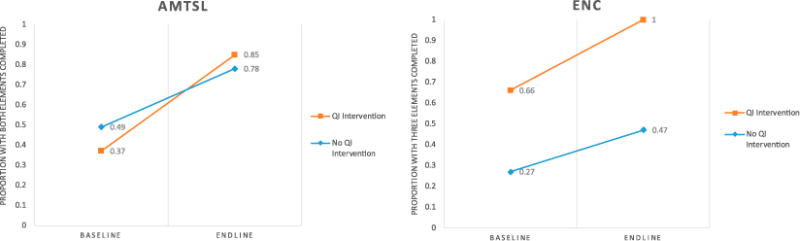
Change within groups between baseline and endline for AMTSL and ENC. North Kivu, DRC 2015–2017

Figure 1 shows the change over time for AMTSL and ENC for the enhanced intervention group and control group. Both groups improved significantly between baseline and endline measures for AMTSL (*p* < .001 for both groups), and for ENC (*p* < .001 enhanced intervention group, *p* = .006 control group). Notably, the percentage of ENC completion was significantly higher at baseline for the enhanced intervention group (*p* < .001), but there was no significant difference between groups at baseline for AMTSL outcomes. In the enhanced intervention group at endline, all newborns had received the three components of ENC.

In the multivariable model (Table 4), the enhanced intervention group had a significantly greater rate of change than the control group for AMTSL (aOR 3.47, 95%CI: 1.17–10.23), controlling for age group, marital status, parity, and schooling.

In the modified GEE model for ENC, the enhanced intervention group had a greater rate of change than the control group (OR: 49.62, 95% CI: 2.79–888.28). Furthermore, both time (OR: 2.44, 95% CI: 1.28–4.66) and group (OR: 5.02, 95% CI: 2.72–9.28) were significant, indicating that completion was higher both at endline overall and among the QI-enhanced group overall (Table 5).

## Discussion

We report improvements in maternal and newborn care delivery in a protracted conflict setting following an enhanced intervention of a QI process after clinical training and provision of supplies. These improvements enhance global health security by facilitating collaborative efforts to achieve core capacities required by global health security frameworks and the WHO. As expected, both groups improved between baseline and endline on the delivery of maternal and newborn care following clinical training and the provision of medical equipment and supplies. Although the enhanced intervention group did not show significantly higher levels of AMSTL completion than the control group, they did improve at a greater rate. Both groups had fairly high completion rates for AMSTL at endline with 85% for the enhanced intervention group and 78% for the control group. The enhanced intervention group also showed a greater rate of change over time for ENC and achieved 100% ENC coverage at endline, which was significantly higher than the control group at 47%. The difference in outcome measures may be due to the relatively new emphasis on ENC and thus the QI was able to have a greater impact. Our findings indicate that the QI process did have an added effect on the provision of maternal and neonatal care beyond what was due to the clinical training.

We believe that the QI process was able to facilitate improved care through changes that addressed improvements in the logistics of work, such as ensuring that a daily supply of oxytocin was taken from the refrigerator and put in the delivery room in a cooler so that it was accessible to the solitary provider and able to be delivered in a timely manner. Another action was to pair matrons who had low literacy with partners who could assist with filling in the partograph. Other actions reinforced the clinical training, such as creating visual aides to put on the facility walls and conducting learning sessions with staff who did not attend the training or those who needed more applied experience to improve. Finally, some actions did not directly apply to the outcomes measures, they addressed other aspects of quality care, such as improving privacy for women during labour.

It is also important to note that while the QI process implemented through this study focused on maternal and neonatal care, there were other additional benefits from the participatory method. Many of these results were not measured by the QI process or this study, but qualitative feedback from the staff and QI coaching team indicate that the impact of the intervention spread beyond the outcomes measured in this paper. For example, health facility staff were empowered to not only identify problems in their facilities, but also to identify solutions and create change in themselves. These skills remain with the staff who can continue to use them beyond the scope of the project for additional improvements within their health facilities. They were trained on the importance of collecting and using data to inform and improve their work. Existing supervisory relationships were strengthened through this process and the skills gained by the supervisors could be utilised with other facility staff outside of labour and delivery.

As this is one of the first times that the QI method has been implemented in a protracted conflict setting, this study also demonstrated that the enhanced intervention can be successfully implemented in this context. Certain aspects of the QI process, such as the scheduling of regular supervision visits due to access and security issues, and incorporating and training new team members as staff changed, did have to be adapted or made more flexible due to the constraints of the setting. Building on existing supervisory systems, maintaining flexibility with scheduling, and planning for the need to incorporate new team members over time due to staff turnover will be essential to the success of QI implementation in other protracted conflict settings. Although the QI process was able to facilitate gains in improved delivery and quality of care beyond the gains seen through clinical training, further research is needed to investigate the level of improvement seen without clinical training, and the feasibility of applying QI in more acute settings where staff turnover and supervision may be even more greatly impacted.

## Limitations

There are several limitations to this study which should be noted. This was a convenience sample of health facilities receiving programmatic support by IMC and therefore not representative of other health facilities in DRC. Our study indicators were measured using face-to-face interviews and data recorded on partographs. Each source has potential biases which may have resulted in under- or over-reporting of items. While we did not use health staff to interview women, because the interviews took place in the health facilities, women may still have been reluctant to report on negative treatment or a lack of treatment. Women’s ability to recall receipt of the AMTSL components may have resulted in estimates that were higher or lower than actual receipt of care. ENC components recorded on the partographs may have been over-reported if staff recorded care that was not delivered, or staff may have forgotten to check off items in the course of delivering care. Additionally, the QI process did include improvement aims related to data quality and record keeping, two areas identified during the baseline data collection period as particularly poor. As the components of the ENC variable were extracted from partographs completed by health facility staff, some of the improvement in ENC delivery could have been due to improved record keeping. Two of the biggest challenges in adapting the QI method to this protracted conflict context were: limited access to health facilities because of remote locations and security issues, and limited communication between project staff, health facilities, and study participants. As a result of these challenges, supervision was not as frequent as might have taken place in a non-humanitarian setting. Finally, because the enhanced intervention group reported delivery of ENC components for 100% of the endline sample, we had to run a simplified GEE model without controlling for sociodemographic variables, resulting in very wide confidence intervals.

## Conclusions

This is one of the first times that this QI methodology has been used in a protracted conflict setting. Both the enhanced intervention group and the control group showed improvements over time following clinical training on BEmONC, ENC, and partograph use. This underscores the importance of the clinical care training provided to facility labour and delivery staff prior to the start of the enhanced intervention. However, as was demonstrated in this paper, the enhanced intervention group demonstrated a significantly greater rate of change in the delivery of AMTSL and ENC beyond the improvements from the clinical training and was able to achieve significantly higher rates of completion of ENC. In humanitarian conflict settings, where resources and external support are particularly scarce, use of a participatory QI method where health facility staff take ownership of the improvement process may provide greater gains in the delivery of quality health care.

## Figures and Tables

**Table 1:** Outcome indicators and data sources

Indicator	Numerator	Denominator	Components	Data source(s)
Percentage of deliveries in facilities with AMTSL^a^	No. of women in facilities who receive oxytocin and uterine massage after delivery of placenta	No. of women who delivered in the facility in the same time period	(1) Oxytocin; (2) uterine massage	PEIs
Percentage of newborns born in facilities who receive essential newborn care (ENC)^b^	No. of newborns that receive three elements of essential newborn care	No. of newborns delivered in the facility during the same time period	(1) Clean cord care; (2) application of antibiotic to eyes; (3) weight	Matched partographs

Note: North Kivu, DRC 2015–2017.

a AMTSL: New WHO Recommendations Help to Focus Implementation. WHO, 2014.

b Newborn Health in Humanitarian Settings Field Guide. UNICEF and Save the Children, New York, 2015.

**Table 2:** Displacement and delivery characteristics of PEI respondents with matched partographs, by time point

	Baseline (*N* = 200)	Endline (*N* = 194)
	
	*n* (%)	*n* (%)

**Displaced status**		
Not displaced	173 (86.50)	110 (56.70)
Displaced	24 (12.00)	84 (43.33)
Missing	3 (1.50)	0 (0.00)

**Length of displacement**	*N* = 24	*N* = 84
Displaced, 2 years or less	15 (62.50)	39 (46.43)
Displaced, more than 2 years	6 (25.00)	41 (48.81)
Missing/do not know	3 (12.50)	4 (4.76)

**Mode of transport to health facility for current delivery**		
By foot	157 (78.50)	159 (81.96)
By motorcycle	41 (20.50)	31 (15.98)
Other	2 (1.00)	3 (1.55)
Missing	0 (0.00)	1 (0.55)

**Location preference for current delivery**		
Current health facility	173 (86.50)	168 (86.60)
Other location	25 (12.50)	26 (13.40)
Do not know	2 (1.00)	0 (0.00)

**Location of most recent prior delivery**	*N* = 170	*N* = 171
This facility	92 (54.12)	82 (47.95)
Another facility	47 (27.65)	63 (36.84)
Home	12 (7.06)	12 (7.02)
Other	16 (9.41)	10 (5.85)
Missing	3 (1.76)	4 (2.34)

Note: North Kivu, DRC 2015–2017.

**Table 3:** Sociodemographic characteristics by time point

	Baseline *n* = 200)	Endline (*n* = 194)	
		
	*n* (%) or *μ*(σ)	*n* (%) or *μ* (σ)	*p*-Value

**Mean age**^a^	25.33 (5.77)	26.8 (6.39)	**0.02**

**Age group**^a^			
18–24	94 (47.47)	76 (39.79)	0.07
25–34	86 (43.43)	84 (43.98)	
35+	18 (9.10)	31 (16.23)	

**Parity**			
Primiparous	30 (15.00)	23 (11.86)	0.36
Multiparous	170 (85.00)	171 (88.14)	

**Mean number of previous births**^c^	4.8 (2.40)	5.3 (2.45)	0.12

**School level**			
None	45 (22.50)	32 (16.49)	0.19
Primary	79 (39.50)	92 (47.42)	
Secondary or greater	76 (38.00)	70 (36.08)	

**Marital status**^b^			
Married	182 (91.00)	172 (89.12)	0.53
Single, not cohabiting	18 (9.00)	21 (10.89)	

Notes: North Kivu, DRC 2015–2017. Bolded values indicate significance at *p* < .05 level.

a Missing two observations with unknown or illogical ages at baseline and three at endline.

b Missing one observation at endline.

c Only assessed for multiparous women.

**Table 4:** GEE outcomes for rate of change of AMTSL, controlling for sociodemographic variables

		AMTSL (n = 376)
		aOR (95% CI)

Time Group	Endline vs. baseline QI intervention vs. no QI intervention	3.04 (1.73–5.34) 0.57 (0.21–1.53)
Rate of change		3.47 (1.17–10.23)
Age group	25–34 vs. 18–24 35+ vs. 18–24	0.98 (0.66–1.46) 1.29 (0.61–2.69)
Marital status	Single not cohabiting vs. married or cohabiting	1.46 (0.62–3.45)
Parity	Primiparous vs. multiparous	0.73 (0.39–1.35)
School level	Primary vs. none Secondary or more vs. none	1.10 (0.60–2.02) 1.29 (0.62–2.67)

Notes: North Kivu, DRC 2015–2017. Bolded values indicate significance at the *p* < .05 level.

**Table 5:** GEE model for rate of change between baseline and endline for ENC

	OR (95% CI)

Endline vs. baseline	2.44 (1.28–4.66)
Intervention vs. control	5.02 (2.72–9.28)
Rate of change	49.62 (2.79–888.28)

Note: Bolded values indicate significance at the *p* < .05 level.
